# An immunohistochemically positive E-cadherin status is not always predictive for a good prognosis in human breast cancer

**DOI:** 10.1038/sj.bjc.6605991

**Published:** 2010-11-09

**Authors:** P Querzoli, D Coradini, M Pedriali, P Boracchi, F Ambrogi, E Raimondi, R La Sorda, R Lattanzio, R Rinaldi, M Lunardi, C Frasson, F Modesti, S Ferretti, M Piantelli, S Iacobelli, E Biganzoli, I Nenci, S Alberti

**Affiliations:** 1 Section of Anatomic Pathology, Department of Experimental and Diagnostic Medicine, University of Ferrara, 44100 Ferrara, Italy; 2 Biostatistics for Bioinformatics and Clinical Translational Research, Institute of Medical Statistics and Biometry, University of Milano, 20133 Milano, Italy; 3 Unit of Cancer Pathology, Department of Oncology and Neurosciences and CeSI, Fondazione ‘G. D' Annunzio’, University of Chieti, 66100 Chieti, Italy; 4 Unit of Medical Oncology, Department of Oncology and Neurosciences and CeSI, Fondazione ‘G. D' Annunzio’, University of Chieti, 66100 Chieti, Italy; 5 Department of Basic and Applied Medical Sciences, University of Chieti, 66100 Chieti, Italy; 6 Fondazione IRCCS, Istituto Nazionale tumori, 20133 Milano, Italy

**Keywords:** E-cadherin, breast cancer, prognosis

## Abstract

**Background:**

In primary breast cancers dichotomic classification of E-cadherin expression, according to an arbitrary cutoff, may be inadequate and lead to loss of prognostic significance or contrasting prognostic indications. We aimed to assess the prognostic value of high and low E-cadherin levels in a consecutive case series (204 cases) of unilateral node-negative non-lobular breast cancer patients with a 8-year median follow-up and that did not receive any adjuvant therapy after surgery.

**Methods:**

Expression of E-cadherin was investigated by immunohistochemistry and assessed according to conventional score (0, 1+, 2+, 3+). Multiple correspondence analysis was used to visualise associations of both categorical and continuous variables. The impact of E-cadherin expression on patients outcome was evaluated in terms of event-free survival curves by the Kaplan–Meier method and proportional hazard Cox model.

**Results:**

Respect to intermediate E-cadherin expression values (2+), high (3+) or low (0 to 1+) E-cadherin expression levels had a negative prognostic impact. In fact, both patients with a low-to-nil (score 0 to 1+) expression level of E-cadherin and patients with a high E-cadherin expression level (score 3+) demonstrated an increased risk of failure (respectively, hazard ratio (HR)=1.71, confidence interval (CI)=0.72–4.06 and HR=4.22, CI=1.406–12.66) and an interesting association with young age.

**Conclusions:**

The findings support the evidence that high expression values of E-cadherin are not predictive for a good prognosis and may help to explain conflicting evidence on the prognostic impact of E-cadherin in breast cancer when assessed on dichotomic basis.

The adhesion of epithelial cells to their neighbours determines cellular and tissue morphology, and regulates some major cellular processes including differentiation, growth and motility. Thus, disruption of normal cell–cell adhesion in transformed cells contributes to tumour cells migration and proliferation, leading to tissue invasion and metastatic spread. Such a disruption can be achieved by downregulating the expression of cadherin family members, in particular E-cadherin, whose importance in maintenance of normal cell architecture is supported by the observation that hereditary predisposition to gastric cancer is associated with germ line mutation in the gene encoding for E-cadherin (Hirohashi, 1998) or that lobular breast carcinomas frequently show mutation of *CDH1* gene with a decreased E-cadherin expression (Qureshi *et al*, 2006). In addition, *in vitro* and *in vivo* experiments have shown that loss of E-cadherin is a crucial step in metastatic spread allowing malignant cells to migrate into the surrounding tissue and to enter blood vessels (Frixen *et al*, 1991; Birchmeier and Behrens, 1994; Kleer *et al*, 2001). Consistently, the downregulation of E-cadherin has been found correlated with short disease-free survival and poor outcome in many tumour from epithelial origin, including breast cancer (Asgeirsson *et al*, 2000; Heimann *et al*, 2000; Berx and Van Roy, 2001; Lim and Lee, 2002; Pedersen *et al*, 2002; Rakha *et al*, 2005; Gould Rothberg and Bracken, 2006). However, some clinical studies do not support this evidence (Lipponen *et al*, 1994; Parker *et al*, 2001), and there are conflicting reports about the usefulness of E-cadherin expression as an independent prognostic marker in invasive breast cancer (Bukholm *et al*, 1998; Howard *et al*, 2004, 2005). In fact, although a low expression of E-cadherin has been found in invasive carcinomas without metastases, high E-cadherin levels have been observed in nodal metastases as compared with their primary tumour (Beavon, 2000; Kowalski *et al*, 2003; Harigopal *et al*, 2005; Jeschke, 2007). In addition, experimental studies have shown that E-cadherin-mediated cell–cell adhesion promotes cell survival protecting tumour cells from chemotherapy and from destruction while in the circulation as tumour cell clumps (St Croix and Kerbel, 1997; Kantak and Kramer, 1998; Gillett *et al*, 2001; Friedl *et al*, 2004). This suggests, on one hand, the inadequacy of the proposed model for a positive association between favourable prognosis and E-cadherin expression and, on the other hand, that immunohistochemical procedures, currently used to quantify E-cadherin expression, might contribute to the inconsistencies leading to erroneous prognostic indication, if not to the loss of prognostic significance for E-cadherin evaluation (Gould Rothberg and Bracken, 2006). Hence, we performed a retrospective study aimed to investigate the prognostic impact of E-cadherin expression, immunohistochemically (IHC) evaluated on a consecutive case series of breast cancers. To better dissect the contribution of E-cadherin expression to prognosis, IHC values were assessed in expression scores instead of dichotomizing them according to a pre-defined cutoff. Our findings show that E-cadherin expression corresponding to 0 to 1+ or 3+ score is associated with an unfavourable prognosis with respect to score 2+. This may help to explain conflicting evidence on the prognostic impact of E-cadherin in breast cancer.

## Patients and methods

### Patients

We considered 234 consecutive patients who underwent surgery for a breast cancer between January 1989 and December 1993 at the Surgical Units of Ferrara S. Anna Hospital University or at Surgical Units of the Ferrara province's hospitals. Informed written consent was obtained from all patients and the study was approved by the University of Ferrara Research Ethics Committee. All patients had a non-lobular cancer and did not receive any adjuvant therapy after surgery because axillary lymph node negative (pN0) at diagnosis. Additional eligible criteria were: pathologic stage T1 to T3, availability of at least 10 resected axillary lymph nodes, absence of synchronous bilateral tumours or any other malignancy before breast cancer diagnosis and up to 6 months after surgery, absence of distant metastases at diagnosis and up to 6 months after surgery, and no neo-adjuvant therapy. Clinical baseline and patients follow-up data (date and site of relapse, last follow-up time, date of death and cause of death) were extracted from the Ferrara Cancer Registry. Data on patient age, tumour histology, pathologic stage, grading, oestrogen receptor (ER) and progesterone receptor status were also collected. After assessment of routine biological markers, for 204 patients (Table 1), a residual paraffin-embedded tissue material of the primary tumour was available for the immunohistochemical evaluation of E-cadherin expression. The protocol of this study was approved by the board of the Ministry of the University and Research (‘*Identification and validation of new markers of metastasizing phenotype of breast cancer’*, prot. MM06095812_006, 2000).

The article was prepared in agreement with the reporting recommendations for tumour marker reporting studies (McShane *et al*, 2005).

### Tissue microarrays (TMAs) and E-cadherin IHC

**Figure 1:**
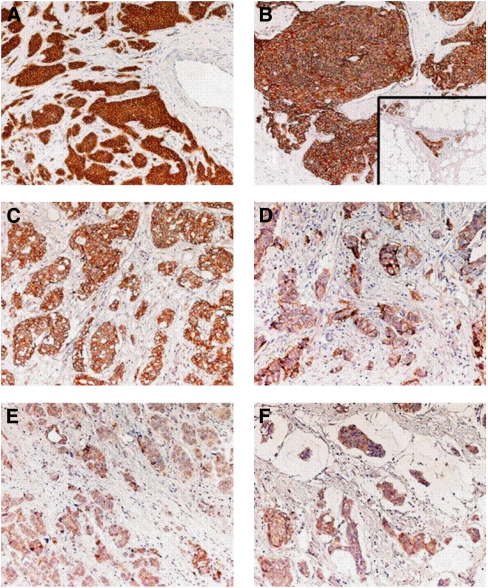
Immunohistochemical analysis of E-cadherin expression breast cancer. (**A**, **B**) Ductal infiltrating breast cancers. Both cases express E-cadherin in almost 100% of the cells, with high, essentially uniform membrane expression levels (3+ **B**) insert: normal breast duct; corresponding E-cadherin expression levels were utilised as internal reference (2+). (**C**, **D**) Ductal infiltrating breast cancers. Both cases express E-cadherin at intermediate levels (2+). However, in the (**C**) case almost 100% of the cells express E-cadherin, whereas a considerable heterogeneity is observed in the (**D**) case. (**E**, **F**) Representative breast cancer cases with low (1+) E-cadherin expression levels. (**E**) Ductal infiltrating breast cancer; (**F**) mucinous-type breast carcinoma.

TMA blocks were assembled as follows. A true-cut needle (4 mm in internal diameter) was used to punch 3-mm spaced holes in the recipient block. Donor blocks of formalin-fixed, paraffin-embedded archival primary tumour samples were retrieved after re-evaluation of haematoxylin- and eosin-stained slides. Representative tumour areas were identified, 4 mm diameter cores of tumour tissues were removed from each donor block and transferred in the recipient block (24 samples per slide). Tissue microarray was then incubated for 15 min at 37 °C to allow the tumour cores to firmly adhere to the recipient block. Consecutive 5-*μ*m thick sections were cut from the TMA and mounted on polarised slides. Slides were deparaffined, rehydrated and treated with 3% H_2_O_2_ in methanol for 10 min to block endogenous peroxidase activity. The slides were processed in a microwave oven in a TEC buffer (Tris–citrate–EDTA), pH 7.8, to unmask antigenic sites after formalin fixation. IHC was performed with an automated immunostainer (Ventana NEXES, Medical System, Tucson, AZ, USA). Slides were stained for E-cadherin using the ECH-6 antibody (Medite, Castelnuovo Del Garda, Italy) and Vectastain ABC peroxidase kit (Vector Laboratories, DBA Italia, Segrate, Italy) was used to reveal antibody binding. Slides treated with normal serum or isotype-matched antibodies were used as negative controls. Endogenous biotin was saturated with a biotin blocking kit (Vector Laboratories). Two pathologists (MP and PQ) independently examined all TMA sections. For each tumour at least 400 cells were counted, and the percentage of immunostained cells was recorded. E-cadherin expression was scored according to the product between the intensity coefficient (0, negative; 1, low; 2, moderate; 3, strong) and the frequency of positivity coefficient (0, no coloured cells; 1, 1–9% 2, 10–49% 3, 50–79% 4, 80–100%) and categorised as follows: 0, negative score; 1+, score 1–4; 2+, score 5–8; 3+, score 9–12. Figure 1 shows some representative examples for different expression levels of E-cadherin.

### Statistical analysis

The association between E-cadherin and other clinicobiological markers, namely patient age, histologic type, grading, pT and ER status was investigated through multiple correspondence analysis (MCA) that visualises on a bi-dimensional plot the association of both categorical and continuous variables (Greenacre, 1994; Marubini and Valsecchi, 1995). For the latter, MCA has the advantage of implying neither linearity nor specific distribution characteristics and to allows to visualise association between markers and tumours. The points in the plot represent the tumours, whereas the markers are labelled according to their category. Points close to each other in a plot correspond to tumours with similar characteristics, whereas close marker labels correspond to associated marker categories. The use of a bi-dimensional plot, easy to interpret, is possible at the expense of loosing some information on the pattern of associations. The distance between points is based on a *χ*^2^ metric, whereas the measure on the axes do not have any physical meaning. To help the interpretation of the results, a cluster analysis was performed using the coordinates of the points, representing tumours, computed by the MCA. The silhouette index (Kaufman and Rousseeuw, 1990) was used to select the number of cluster to be considered.

As reported in Table 1 biological variables were categorized according to conventional cutoff or score except for patient age that was subdivided according to physiological-related criteria (⩽40 years, young patients; 41–50 years, pre-menopausal patients; 51–55 years, peri-menopausal patients; 56–70 years, post-menopausal patients; >70 years elderly patients). Cribriform, mucinous, papillary and tubular histologies were grouped together as ‘other histology’. To evaluate the effect of E-cadherin on patient outcome, the end point of the survival analysis was the time elapsed from surgery to the occurrence of the first adverse event (e.g., local relapse, distant metastasis, contralateral tumour, a second tumour and death without evidence of neoplastic disease). Event-free survival curves were plotted by the Kaplan–Meier method and a proportional hazard Cox model was applied (Walter *et al*, 1987). In the multivariable model, E-cadherin effect was adjusted for grading, pT, age (as continuous variable) and ER (10% cutoff). Grading categories were coded as two indicators variables as defined by Walter allowing the comparison between contiguous levels (Schoenfeld, 1982). Because only two patients were T3, pathological stages T2 and T3 were grouped together. To evaluate the appropriateness of proportional hazard Cox model assumption Schoenfeld residuals were analysed (Hazan *et al*, 2000). Hazard ratios (HRs) with 95% confidence intervals (CIs) were used to quantify the prognostic impact of variables. R software (http://www.r-project.org) was utilised throughout this study.

## Results

### Association analysis

**Figure 2:**
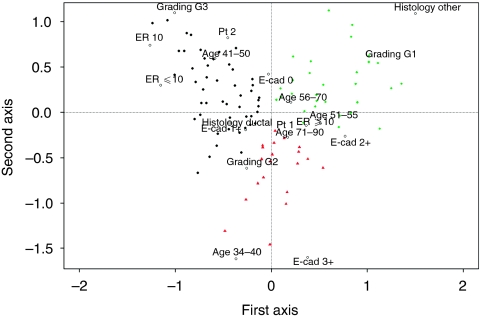
Association among E-cadherin (E-cad), oestrogen receptor (ER), histologic type, grading, pathological stage (pT) and age. Multiple correspondence analysis plot shows the projections of the categories of E-cadherin (score 0, 1+, 2+, 3+), ER (0, ⩽10, >10%), histologic type (ductal, other), grading (G1, G2, G3) and age (34–40, 41–50, 51–55, 56–70, 71–90). Categories of the variables close to each other correspond to associated marker categories and clinical characteristics. The coloured points represented in the plot correspond to the 234 tumours included in the study. Tumours with similar profiles are projected near to each other. The profiles are described by the position of the different marker categories. Moreover, to help interpreting the plot, a cluster analysis was performed to highlight tumours with similar profiles. Three clusters were chosen using specific indices and the points coloured accordingly.

Figure 2 shows the plot from the MCA analysis in which the first two factorial axes explain 95% of the total variability (first axis: 64% second axis: 28%). The horizontal axis separates, on the right, G1, ER>10%, ‘other histology’, post-menopaused patients and E-cadherin 2+ whereas, on the left, low values of ER, G3 and pre-menopaused patients. The vertical axis mainly separates E-cadherin score 0 and 3+. Cluster analysis, performed on the results of the MCA, highlighted three clusters of patients, represented in the plot by different colours. The green cluster is characterized by patients with some favourable characteristics (low tumour histologic grade, and peri-/post-menopausal status). The black cluster is characterized by pre-menopausal patients having a tumour associated with less favourable characteristics: low ER values and high histologic grade. The red cluster is mainly composed of young and elderly patients, having a tumour characterized by an intermediate histologic grade. E-cadherin 1+ was associated with the black cluster, whereas E-cadherin 3+ was associated with the red cluster. E-cadherin 2+ was associated with both green and red clusters while E-cadherin 0 was associated with both red and black clusters. Noteworthy, E-cadherin 3+ category was plotted near young patients category. Although this result is based on a low number of patients in E-cadherin 3+ and 34–40 age categories, it suggests a possible interesting association between high E-cadherin levels and more aggressive disease.

### Event-free survival analysis

**Figure 3:**
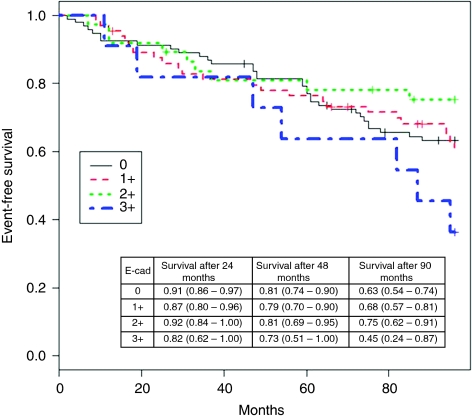
Kaplan–Meier event-free survival curves stratified according to the expression level of E-cadherin (E-cad). Patients with low-to-nil (score 0 to 1+) or a high (score 3+) E-cadherin expression show an increased risk of relapse with respect to patients with an intermediate E-cadherin expression level (score 2+).

The follow-up of the study was closed on the 31 December 2002. The median follow-up of the 204 patients was 118 months even though it was curtailed at 8 years when only about 12% patients were lost to follow-up. During follow-up, 23 patients developed distant metastases, 14 a local relapse, 8 a contralateral tumour, 8 another malignancy and 37 dead as first event. The Kaplan–Meier survival estimates, according to the different scores of E-cadherin, are shown in Figure 3. The HR for E-cadherin 1+ *vs* E-cadherin 0 was 1.04 (*P*=0.88), for E-cadherin 2+ *vs* E-cadherin 1+ was 0.62 (*P*=0.22) and for E-cadherin 3+ *vs* E-cadherin 2+ was 2.94 (*P*=0.03). The results of the multivariable model are reported in Table 2 where E-cadherin 0 and 1+ are grouped together. Patients whose tumour had an E-cadherin expression low-to-nil (score 0 to 1+) showed a tendency toward an increased risk of failure *vs* patients with intermediate E-cadherin expression (score 2+ HR=1.71; CI=0.72–4.06). Noteworthy, also patients whose tumour expressed high levels of E-cadherin (score 3+) had an unfavourable outcome when compared with patients with E-cadherin 2+ (HR=4.22; CI=1.41–12.66). The prognostic impact of the other variables analyzed, for example, a lower risk of failure associated with ER expression, corresponded to that expected from previous studies.

## Discussion

Downregulation of the epithelial cell–cell adhesion molecule E-cadherin is frequently associated with neoplastic transformation and progression in breast cancer (Asgeirsson *et al*, 2000; Heimann *et al*, 2000; Berx and Van Roy, 2001; Lim and Lee, 2002; Pedersen *et al*, 2002; Rakha *et al*, 2005; Gould Rothberg and Bracken, 2006). However, there are conflicting reports about the usefulness of E-cadherin status as an independent prognostic marker in invasive breast cancer (Lipponen *et al*, 1994; Bukholm *et al*, 1998; Parker *et al*, 2001; Howard *et al*, 2004, 2005). Aim of this study was to assess the relationship between E-cadherin expression levels and patient outcome. Our findings provide evidence that, with respect to intermediate values (score 2+), low-to-nil (score 0 to 1+) or high (score 3+) E-cadherin expression values are associated with an unfavourable prognosis explaining, at least in part, the conflicting clinical evidence on the prognostic impact of E-cadherin when assessed on dichotomic basis.

One of the possible biological explanations of such a finding could be the epithelial–mesenchymal transition, a process in which epithelial cells lose their characteristic polarity, disassemble cell–cell junctions and become more migratory. One of the most peculiar aspects of epithelial–mesenchymal transition is the ‘cadherin isoform switching’ that consists in a downregulation of E-cadherin expression and a concomitant upregulation of N-cadherin with profound effects on cell phenotype and behaviour. In fact, upregulation of N-cadherin has been shown to promote tumour cell motility and invasion (Cavallaro *et al*, 2002), according to the ‘cadherin switching’ triggered during normal embryonic development (Christofori, 2003; Nakajima *et al*, 2004). However, the term ‘cadherin switching’ that usually refers to a switch from E-cadherin to N-cadherin expression, it also includes situations in which E-cadherin expression levels do not change significantly from the normal, and cells increase expression of N-cadherin or co-express inappropriate cadherins including R-cadherin, cadherin-11 and even P-cadherin altering tumour cells behaviour towards a more invasive phenotype (Nieman *et al*, 1999; Tomita *et al*, 2000; Stefansson *et al*, 2004; Paredes *et al*, 2005). In fact, it has been reported that epithelial cells expressing significant amounts of E-cadherin and just a small amount of N-cadherin, increase their motility and that a forced expression of N-cadherin in non-invasive, E-cadherin-positive cells may produce invasive cells, even though they continue to express high levels of E-cadherin (Nieman *et al*, 1999). For example, Hazan *et al* (2000) have shown that the non-metastatic breast cancer cell line MCF7 can be transformed in a metastatic cell line when transfected with N-cadherin and that when injected into the mammary fat pad of nude mice, these N-cadherin-expressing cells metastasised to visceral organs whereas control MCF7 cells did not. This suggests that N-cadherin may activate cell motility even in the presence of E-cadherin, resulting in a more aggressive and invasive tumour phenotype. Unfortunately, we were unable to verify such an interesting biological hypothesis on our case series because of the dramatic reduction of leftover material that should bias the results.

In conclusion, present results indicate that the IHC evaluation of E-cadherin expression in terms of negative/positive status should perhaps be too naive as E-cadherin positivity might actually hide complex biological interactions that may determine the course of tumour progression and disease outcome. A hypothesis that certainly deserves further investigation.

## Figures and Tables

**Table 1:** Clinicopathological characteristics of node-negative breast cancer patients with available leftover material for E-cadherin evaluation

**Categorical variables**	***N***	**%**
*Age, n*=*204*		
34–40	12	5.88
41–50	45	22.06
51–55	14	6.86
56–70	85	41.67
71–90	48	23.53
		
*Histologic type, n*=*204*		
Cribriform	5	2.45
Ductal	167	81.86
Mucinous	12	5.88
Papillar	5	2.45
Tubular	11	5.39
Medullar	4	1.97
		
*pT stage, n*=*204*		
pT1	159	77.94
pT2	43	21.08
pT3	2	0.08
		
*Histological grade, n*=*204*		
G1	50	24.51
G2	117	57.35
G3	37	18.14
		
*Oestrogen receptor, n*=*172*		
0%	16	9.30
1–10%	24	13.95
>10%	132	76.75
		
*Progesterone receptor, n*=*169*		
0%	14	8.28
1–10%	34	20.12
>10%	120	71.60
		
*E-cadherin, n*=*204*		
0	92	45.10
1+	64	31.37
2+	37	18.14
3+	11	5.39

Abbreviation: pT stage=pathological stage.

**Table 2:** Risk analysis for event-free survival in a multivariate Cox model

**Variable**	**Coefficient estimate**	**HR**	**95% CI**	***P*-value**
E-cadherin 0–1+ *vs* 2+	0.54	1.71	0.722–4.062	0.22
E-cadherin 3+ *vs* 2+	1.44	4.22	1.406–12.664	0.01
pT2 *vs* pT1	0.36	1.44	0.773–2.664	0.25
G2 *vs* G1	0.03	1.03	0.567–1.880	0.92
G3 *vs* G2	0.58	1.78	0.756–4.192	0.19
Age	0.05	1.05	1.027–1.072	<0.001
ER >10 *vs* <10	−0.71	0.49	0.261–0.934	0.03

Abbreviations: CI=confidence interval; ER=oestrogen receptor; HR=hazard ratio.
